# Comparison of blood and urine nephrin levels in preeclampsia and intrauterine growth retardation

**DOI:** 10.12669/pjms.321.8865

**Published:** 2016

**Authors:** Fatma Ozdemir, Ahter Tanay Tayyar, Gokhan Acmaz, Huseyin Aksoy, Gozde Erturk, Sabahattin Muhtaroglu, Mehmet Tayyar

**Affiliations:** 1 Fatma Ozdemir, MD, Department of Obstetrics and Gynecology, Education and Research Hospital, Kayseri, Turkey; 2 Ahter Tanay Tayyar, MD, Department of Obstetrics and Gynecology, Private Erciyes Hospital, Kayseri, Turkey; 3 Gokhan Acmaz, MD, Department of Obstetrics and Gynecology, Education and Research Hospital, Kayseri, Turkey; 4 Huseyin Aksoy, MD, Department of Obstetrics and Gynecology, Military Hospital, Kayseri, Turkey; 5 Gozde Erturk, Department of Biostatistics, Erciyes University Faculty of Medicine, Kayseri, Turkey; 6 Dr. Sabahattin Muhtaroglu, Professor, Department of Biochemistry, Erciyes University Faculty of Medicine, Kayseri, Turkey; 7 Dr. Mehmet Tayyar Professor, Department of Obstetrics and Gynecology, Erciyes University Faculty of Medicine, Kayseri, Turkey

**Keywords:** Preeclampsia, Intrauterine growth retardation, Serum Nephrin, Urine Nephrin

## Abstract

**Objective:**

To evaluate the relation between nephrin levels and preeclampsia severity by comparing serum and urine levels of nephrin in the severe and mild groups according to severity of associated intrauterine growth retardation (IUGR) development.

**Methods:**

A total of 150 patients who attended our ante-natal clinic (ANC) were included in this study. We had 5 groups; Group 1:30 patients with mild preeclampsia (MP) and normal fetal development (NFD), Group 2:30 patients with severe preeclampsia (SP) and NFD, Group 3: 30 patients with MP and IUGR, Group 4: 30 patients with SP and IUGR and Group 5: 30 volunteers who were normotensive and non-preeclamptic. We obtained both blood and urine samples for measuring nephrin levels.

**Results:**

Both serum and urine nephrin levels were significantly higher for the fourth group compared with all other groups (p<0.001). The levels of SP group with NFD were measured considerably higher than MP group out of IUGR and control group (p<0.001). Urine and serum nephrin levels with gestational age of delivery showed a negative correlation (r=-0.621, p<0.001) and also urine and serum nephrin levels with birth weight showed a negative correlation too (r=-0.655 p<0.001).

**Conclusion:**

Both serum and urine nephrin levels correlated with the severity of preeclampsia and IUGR development.

## INTRODUCTION

Preeclampsia has impact both on perinatal and maternal morbidity and prevalence is 5–7% of all pregnancies. Additionally, systemic inflammatory response leading to generalized endothelial cell dysfunction and inadequate trophoblast invasion of spiral arteries contribute to the spectrum of the disease such as IUGR development.^1^-^3^ IUGR can be defined as a fetus whose weight is under the 10th percentile than expected according to the gestational age and sex, as determined by population standards.^4^

It has been shown that nephrin defect between the podocite feet leads to proteinuria in finnish type congenital nephritic syndrome. This situation means that nephrin is important for the diaphragm integrity and renal filtration capacity.^5^

This study aimed at investigating the importance and effect of endothelial dysfunction on IUGR and preeclampsia etiopathogenesis comparing preeclamptic patients both suffering from IUGR and with NFD by the aid of measured levels of urinary excretion and serum concentration of nephrin. In addition, the objective of this study was to evaluate the relation between the nephrin levels and preeclampsia severity by comparing the levels of nephrin in the severe and mild groups.

## METHODS

The study was approved by the Erciyes University ethics committee (No:2013/125) and all participants signed an informed consent form for the study. This study was conducted in Obstetrics and Gynecology Department of the Erciyes University Faculty of Medicine between February 2013 and January, 2014.

Diagnosis of preeclampsia was made according to the criteria agreed by the National High Blood Pressure Education Program Working Group of National Institutes of Health (NIH) in 2000.^6^ Preeclampsia was defined as blood pressure (BP) of at least 140/90 mmHg after 20 weeks gestation on at least two occasions 6 hours apart, when the absence of gestational trophoblastic disease or multiple pregnancies was confirmed by ultrasonographic examination, with proteinuria more than 0.3 g per 24 hours. BP was measured with a calibrated aneroid sphygmomanometer in the supine position after five minutes rest. Absolute diastolic BP of ≥ 110 mmHg and proteinuria (≥ 2+ [100mg/dL] on a chateterized specimen was diagnostic for SP at admission. Additionally, following criteria with patients considered to have SP; 5g proteinuria in 24 hour urine test, blurred vision, pulmonary edema, epigastric pain, nausea, vomiting, cerebral disorder, thrombocitopenia< 100.000, creatinine level > 0.9 mg/dl, elevated liver enzymes, and hemolysis.

Women who had preeclampsia before were not eligible to be controls. Patients with multiple pregnancies, with chronic renal and vascular disease, diabetes mellitus, patients with fetal anomaly and urinary infection or previous thromboembolic complications were excluded and women who are on anticoagulant therapy or having preeclampsia superimposed on chronic hypertension were not included in the study. None of the patients or controls was in labor at the time of sampling.

The most common sonography-based definition of IUGR is a weight below the 10th percentile for gestational age, although other definitions employing a variety of criteria have been advocated. When a small fetus is detected, it can be difficult to distinguish between the fetus that is constitutionally small versus growth restricted.^7^ Therefore, we applied doppler examination for discriminating IUGR and small for gestational age (SGA).

Gestational age was based on the precise date of the last period and ultrasound measurement of the crown-rump length in the first trimester. Each subject underwent a comprehensive examination for age, parity, length, weight (before and during pregnancy, additionally at the time of labor), gestational age, BP, proteinuria with uristix examination, 24 hour protein value of the urine, haemoglobin, haematocrit, platelet, blood urea nitrogen (BUN), creatinine value and liver function tests such as alanine aminotransferase, aspartate amino-transferase. All volunteers were examined with doppler by the same author (FO).

Fetal biometric measurement, amniotic fluid index, umbilical and middle cerebral artery doppler examination were recorded. Birth weight, gestational age, type of delivery were noted as well.

We obtained both 10 cc blood and 10 cc urine for measuring nephrin levels. Both urine and blood specimens were collected and frozen at -70°C immediately after concentration by centrifugation (5000rpm/10 minutes). Urine nephrin concentrations were measured with a commercially available enzyme-linked immunosorbent assay (Product Name: Human Nephrin (NPHN) ELISA Kit. Brand/Origin: SRB/Shanghai).

A total of 150 patients who were introduced to our clinic were included in this study. We had 5 groups; Group 1:30 patients with MPand NFD, Group 2: 30 patients with SP and NFD, Group 3: 30 patients with MP and IUGR, Group 4: 30 patients with SP and IUGR and Group5: 30 volunteers who were normotensive and non-preeclamptic.

All statistical analysis was performed using SPSS 15. The confidence interval was 95%, and p< 0.05 was considered to be statistically significant. Shapiro–Wilk’s test was used to assess the data normality. Pearson’s correlation analysis was used to compare categoric variables. Values were expressed as mean±standard deviation (SD). The Kruskal- Wallis test was used to compare the nonparametric variables. Spearman corelation analyses were used to indentify relationship between quantitative variables and groups. Dunns test statistics were used for post hoc analyse.

## RESULTS

All groups were homogenous for age, gravidy, parity, abortus, gestational week during diagnosis. Demographic features and laboratory findings of the groups were illustrated in Table I and II. Also, serum and urine nephrin values and correlation of these parameters with BP, birth weight and serum creatinine were showed in Table III and IV.

**Table-I:** Demographic features of the groups.

Features*	Patients with MP and NFD	Patients with SP and NFD	Patients with MP and IUGR	Patients with SP and IUGR	Control group	P value
Age (year)	28 (23.2-32.7)	28 (24-35)	28 (24,5-31)	30.5 (22-34)	26 (25-29)	0.665
Before pregnancy BMI (kg/m^2^)	26.7^a^ (24.5-30)	26.1 (23.9-28.7)	25.6 (21.3-31.3)	24.8 (22.5-27.6)	23.4 (21.5-26.8)	0.025
During pregnancy BMI (kg/m^2^)	32.5^a^ (28.9-36.3)	30.8 (28.8-35.2)	30.5 (25.8-34.7)	30.5 (28.4-34.1)	27.4 (25.4-30.8)	<0.001
During labor BMI (kg/m^2^)	33.8^a^ (30.3-36.6)	31.8 (30 -35.2)	31.15 (27.2-35.3)	30.975 (28.4-34)	28.95 (27.05-32)	0.003
Gravidy	2 (1-3)	3 (1-3)	2 (2-3)	1 (1-3)	2 (1-3)	0.391
Parity	1 (0-1.75)	1 (0-2)	1 (0-2)	0 (0-2)	1 (0-1)	0.612
Abortion	0 (0-1)	0 (0-1)	0 (0-1)	0 (0-0)	0 (0-0)	0.497
Gestational week during diagnosis	35.14 (31.9-36.6)	32.57 (30.6-36.1)	31.14 (29.3-35)	31.5 (30-35.3)	34.21 (30.7-37)	0.055

* All criterias were calculated by using Kruskal-Wallis. All values were expressed as mean and 25-75 percentile.

a) means that when a data matched with control group p<0.001

**Table-II:** Blood pressure and laboratory findings.

Criteria^*^	Patients with MP & NFD	Patients with SP & NFD	Patients with MP & IUGR	Patients with SP & IUGR	Control group	P value
Systolic blood pressure (mmHg)	140^a^ (140-150)	160^a,b,c^ (150-170)	140^a^ (140-150)	160^a,b,c^ (150-160)	100 (100-110)	<0.001
Diastolic blood pressure (mmHg)	90^a^ (90-100)	110^a,b,c^ (100-110)	90^a^ (90-100)	105^a,b,c^ (100-110)	60 (50-70)	<0.001
Urine microprotein/creatinin ratio	0.730^a^ (0.505-1.325)	4.2^a,b,c^ (2.9-8.7)	0.723^a^ (0.499-1.35)	4.7^a,b,c^ (3.2-9.1)	0.158 (0.14-0.18)	<0.001
Urine protein with stick (+)	1 (1-2)^a^	3 (3-3)^a,b,c^	1 (1-2)^a^	3 (3-3)^a,b,c^	0 (0-0)	<0.001
24 h urine protein (gr/dl)	0.911^a^ (0.61-1.3)	6.4^a,b,c^ (5.8-7.7)	1.5^a^ (0.79-2.3)	5.6^a,b,c^ (4.5-7.8)	0.19 (0.13-0.2)	<0.001
Serum creatinin (mg/dl)	0.6 (0.6-0.7)	0.7^a,b,c^ (0.6-0.8)	0.6 (0.6-0.7)	0.7^a,b,c^ (0.7-0.8)	0.6 (0.6-0.7)	<0.001

* All criterias were calculated by using Kruskal-Wallis.

a) means that when a data matched with control group p<0.001.

b) means that when a data matched with MP and NFD group p<0.001

c) means that when a data matched with MP and IUGR group p<0.001

**Table-III:** Serum and urine nephrin values.

Criteria^*^	Patients with MP & NFD	Patients with SP & NFD	Patients with MP & IUGR	Patients with SP & IUGR	Control group	P value
Serum nephrin values (ng/ml)	2.187 (1.679-2.518)	4.285^a,b^ (3.819-5.160)	3.412^a^ (3.012-3.965)	7.411^a,b,c,d^ (6.642-8.478)	0.895 (0.555-1.312)	<0.001
Urine nephrin values (ng/ml)	6.479 (5.575-7.22)	12.035^a,b^ (11.448-12.76)	9.591^a^ (8.99-10.045)	18.385^a,b,c,d^ (17.002-20.23)	2.728 (1.67-3.728)	<0.001

* All criterias were calculated by using Kruskal-Wallis.

a) means that when a data matched with control group p<0.001

b) means that when a data matched with MP and NFD group p<0.001

c) means that when a data matched with SP and NFD group p<0.001

d) means that when a data matched with MP and IUGR group p<0.001

**Table-IV:** Correlation of serum and urine nephrin values to BP, birth weight and serum creatinine.

Criteria	Systolic BP		Diastolic BP		Serum creatinine		Gestational week		Birth weight	
	R	P	R	P	R	P	R	P	R	P
Urine nephrin	0.769	<0.001	0.770	<0.001	0.474	<0.001	-0.621	<0.001	-0.655	<0.001
Serum nephrin	0.752	<0.001	0.749	<0.001	0.459	<0.001	-0.613	<0.001	-0.642	<0.001

r= pearson correlation coefficient.

## DISCUSSION

Our study demonstrates that both serum and urinary nephrin is related with SP and IUGR development. This is perhaps the first study which reports an association between IUGR and urine and serum nephrin levels. It has been claimed that nephrin may be useful marker for SGA prediction during the first trimester; however, it may be useful for the detection of preeclampsia, only in the third trimester.^8^

It has been known that nephrin is expressed in the slit diaphragm between the podocyte foot processes which has a critical role in the maintenance of slit pore integrity and renal filtration capacity. Some studies show that nephrin gene mutations lead to congenital nephritic syndrome of the Finnish type.^9^

It is suggested that nephrin makes a zipper-like structure that acts as a size and charge-selective filtration barrier. Moreover it has signalling functions, capable of regulating podocyte cell polarity, cell survival, and cytoskeletal organization.^10^ It has been found that nephrin may be a marker of subclinical renal damage, can be detected before overt proteinuria and the full clinical features of preeclampsia develops.^5^

We found that both serum and urine nephrin levels were highest in SP and IUGR groups. Morover SP and NFD group had considerably elevated serum and urine nephrin levels. MP and IUGR group had higher serum and urine nephrin levels than control group.

Pearson analyses showed that elevated nephrin levels negatively correlated with birth weight and gestational week and positively correlated with serum creatinine, systolic and diastolic BP.

We are of the opinion that increasing levels of nephrin is related to preeclampsia severity and IUGR development. Although previously reported one study revealed a positive correlation of nephrin with small for gestational age fetuses, this is the first study in literature which illustrated positive correlation between serum-urine nephrin levels and IUGR on the basis of preeclampsia.

This study will open new horizons for the new researchers to investigate nephrin as a diagnostic tool for IUGR development and understanding severity of preeclampsia.

### Limitations of the study

There is a need for further, larger scale studies, interacting with other systemic disorders related to preeclampsia.
